# Predictive Factors for Diabetes Insipidus and Syndrome of Inappropriate Secretion of Antidiuretic Hormone After Transsphenoidal Surgery for Pituitary Adenoma

**DOI:** 10.7759/cureus.116176

**Published:** 2026-09-13

**Authors:** Raghad A Alzahrani, Mohammed A Alyousef, Lamar A Alghamdi, Renad M Alamoudi, Jana M Banjer, Nawaf S Alghamdi, Basil A Alzahrani

**Affiliations:** 1 Otolaryngology - Head and Neck Surgery, King Abdulaziz Medical City, Jeddah, SAU; 2 Faculty of Medicine, King Abdulaziz University, Jeddah, SAU; 3 Internal Medicine, King Abdulaziz Medical City, Jeddah, SAU

**Keywords:** diabetes insipidus, hypertension, hypopituitarism transsphenoidal surgery, pituitary adenoma, siadh, smoking, transsphenoidal surgery

## Abstract

Objective

The objective of this study is to determine preoperative predictors of diabetes insipidus (DI) and syndrome of inappropriate secretion of antidiuretic hormone (SIADH) after transsphenoidal surgery (TSS) for pituitary adenomas. DI and SIADH are common complications associated with TSS.

Methods

This small unicentric retrospective study was conducted in a tertiary referral hospital in Saudi Arabia. The study enrolled 42 patients. We reviewed the medical records of all patients who underwent surgical resection of pituitary adenoma at our hospital between January 2010 and December 2022. The results of urine and serum analyses were collected to establish a diagnosis. Pre- and post-surgery sodium differences were categorized by the pre-surgery value to calculate the percent change in sodium levels. The tumor function was confirmed using histopathology, and radiological reports were reviewed for the size, location, extent, invasiveness of the tumor, and cerebrospinal fluid (CSF) leaks.

Results

Among the 42 patients included, seven (16.7%) had DI and two (4.8%) had SIADH. Two-thirds underwent resection using the microscopic approach, and the remaining patients underwent endonasal TSS. Approximately 43% of patients had functioning pituitary adenomas, with prolactin-secreting adenomas being the most common (21.4%). Three-quarters of the tumors were macroadenomas. No significant between-group differences were observed in the length of hospital stay, preoperative sodium levels, and percentage change in serum sodium (P>.05). Smoking and chronic hypertension were not associated with DI or SIADH. The number of patients with panhypopituitarism (P<.01) or hypothyroidism (P<.01) was significantly different between the DI and no DI groups. However, no factor significantly predicted the development of DI. No significant differences were observed between the SIADH and non-SIADH groups.

Conclusion

The risk of DI increases in patients who develop panhypopituitarism, including central hypothyroidism after surgery. However, no factor reliably predicts the development of DI or SIADH after TSS.

## Introduction

Antidiuretic hormone (ADH) is synthesized in the hypothalamus, transported via the hypothalamic-hypophyseal tract to the posterior pituitary, and stored for later release [1]. Abnormal secretion of ADH is one of the most frequent postoperative complications in patients who undergo pituitary surgery (PS) [2]. Owing to reduced ADH function in cases of diabetes insipidus (DI), patients pass large amounts of diluted urine, resulting in fluid and electrolyte imbalance. This complication affects 2% to 54% of the patients [3]. Syndrome of inappropriate secretion of antidiuretic hormone (SIADH) is the primary cause of hyponatremia after PS. According to several studies, its incidence ranges from 1.8% to 35% [4,5]. Patients with untreated DI and SIADH may experience fluctuations in serum osmolarity and sodium levels, which in extreme situations may result in dehydration and severe mental and neurological sequelae [6-8].

The median hospital stays for patients with normal sodium balance, hypernatremia, and hyponatremia are five, six, and 11 days, respectively [9]. The potential factors for DI include intraoperative cerebrospinal fluid (CSF) leak, younger age, serum sodium fluctuations before and after surgery, large tumor size, and suprasellar extension [10-14]. Furthermore, the underlying pathology is an important predictor of both the occurrence and permanence of postoperative DI [15]. Potential predictors for SIADH include age, tumor size, blood sodium levels before and after surgery, and Cushing’s disease [9,12].

However, no reliable risk factors or markers are currently available to predict the onset of DI or SIADH [16,17]. Several potential risk factors, such as smoking, body mass index (BMI), and hypertension, have not been considered but may be important in predicting the likelihood of postoperative electrolyte imbalance [12]. Over the last six decades, microscopic transsphenoidal surgery (TSS) has been the standard approach for dissecting pituitary tumors. In recent years, endoscopic endonasal surgery has been increasingly used [18]. In this study, we elaborated on these approaches. Unlike previous studies that included various other pituitary pathologies, our study specifically focused on pituitary adenomas. Furthermore, our research adds to the existing literature by exploring potential risk factors, including smoking and hypertension, which have not been fully investigated. Our primary objective was to define the risk factors for and frequency of DI and SIADH after pituitary adenoma resection.

This work was previously presented as an abstract at the American Association of Neurological Surgeons 2020 Annual Scientific Meeting on April 27, 2020.

## Materials and methods

Study design and setting

This small unicentric retrospective study was conducted in the neurosurgery department of a referral center and tertiary academic institution in Jeddah, Saudi Arabia, between January 2010 and December 2022. All procedures were performed in compliance with relevant laws and institutional guidelines, and the study was approved by the relevant institutional review board (HA-02-J-008) on March 27, 2023. The need for informed consent was waived because this was a retrospective study of anonymized data.

Sample size and sampling procedure

All patients with pituitary adenomas who underwent resection at our institution during the study period were enrolled and retrospectively evaluated through their electronic medical records. Patients with Roethke’s cleft cyst, craniopharyngioma, chordoma, or missing data were excluded.

Laboratory test data were collected from the registry to determine the development of DI and SIADH during the hospital stay and the follow-up period. A standard monitoring protocol was followed for all patients in the neurosurgical intensive care unit (ICU) after pituitary adenoma excision, including measurement of serum and urine parameters twice daily throughout the ICU stay. Hourly urine output, urine sodium and osmolarity every 4 h, and serum sodium every 2-4 h of patients who experienced severe hyponatremia (<125 mmol/L) were routinely measured. Volumetric contrast-enhanced magnetic resonance imaging at a field strength of 3 Tesla was used to scan the pituitary gland preoperatively and six weeks postoperatively as the standard of care for all patients. Furthermore, a scan was conducted immediately after surgery if complications were suspected. Tumors were classified as microadenoma and macroadenoma if the tumor size was <1 cm and ≥1 cm, respectively. Fixed criteria were applied to all the patients to confirm the occurrence of DI.

The diagnosis of DI could also be made by an endocrinologist. SIADH was defined by a serum sodium level of <135 mEq/L, plasma osmolarity of <280 mOsmol/kg, urine osmolarity of >100 mOsmol/kg, and urine sodium level of >40 mEq/L. Radiological reports were reviewed to determine the size, location, extent, and invasiveness of the tumor, as well as the presence of CSF leaks.

All patients were preoperatively screened for endocrinopathies. Physician notes were reviewed to determine the presenting symptoms and any development of visual symptoms. Baseline laboratory data for thyroid stimulating hormone (TSH), prolactin (PRL), urine specific gravity, and sodium levels were collected. Preoperative serum sodium was defined as the most recently available venous laboratory result typically obtained 12-24 hours prior to surgery, and postoperative serum sodium was defined as the first venous serum sodium result obtained typically two to four hours after surgery as part of routine inpatient monitoring. The percentage change in serum sodium was calculated by determining the change from the preoperative and postoperative values and dividing that value by the preoperative value and then multiplying by 100. This was done as a normalized value of relative change to allow a comparison among patients with different levels of serum sodium.

Statistical analysis

Microsoft Excel 2016 (Microsoft, Redmond, WA) was used for data collection. Categorical and continuous variables were compared using the Chi-square and independent t-tests, respectively. The Chi-square and Fisher’s exact tests were used to investigate the relationship between potential risk factors and the occurrence of postoperative SIADH and DI. Multiple logistic regression analysis was also used to determine the odds ratios (ORs) of predictive factors in the development of SIADH and DI after operation. Due to the low sample size and incidence rate of the complications, especially SIADH, multivariate analysis was regarded as exploratory in nature taking due caution to avoid any form of overfitting. Statistical significance was defined as a P-value of <0.05 or a 95% confidence interval (CI) that does not include a null value of 1.0. All data analyses were performed using IBM SPSS Statistics version 26.0 (IBM Corp, Armonk, NY, USA) and RStudio version 4.3.1 (RStudio, Public Benefit Corporation (PBC), Boston, MA, USA).

## Results

Diabetes insipidus was observed in seven (16.7%) out of 42 patients. SIADH was noted in two patients (4.8%). Furthermore, 24 patients were women (57.1%). Three patients (7.1%) were active smokers. Visual symptoms were documented in 36 out of 42 patients, and 24 patients (66.7%) complained of visual symptoms. Similarly, headache status was documented in 35 out of 42 patients, in which 17 patients (48.6%) experienced headache. Macroadenoma was the most frequent tumoral entity, affecting 31 patients (73.8%). Thirty-five patients (83.3%) underwent initial surgery, of whom 25 (71.4%) received a gross total resection (GTR). A microscopic transsphenoidal approach was used in 26 patients (61.9%), while an endonasal approach was used in 16 patients (38.1%). Functioning pituitary adenomas were diagnosed in 18 patients (42.9%); hormone-secreting subtype was specified for 22 patients, including nine prolactin-secreting adenomas, four adrenocorticotropic hormone (ACTH)-secreting, six follicle-stimulating hormone (FSH)-secreting, and three luteinizing hormone (LH)-secreting adenomas. The functioning adenoma subtypes relate to a total of 18 functioning adenomas. The baseline characteristics of the studied population are presented in Table 1.

**Table 1:** Distribution of studied patients according to their demographics; smoking status; comorbidities; associated symptoms; length of hospital stay (LHS); and pre-operative, operative, and post-operative clinical data (N=43) Note: Percentages for visual symptoms are calculated based on 36 patients with documented status; for headache, based on 35 patients with documented status; and the secreting hormone subtype exceeds the reported functioning adenoma total (n=18) based on  data available for 22 patients. This discrepancy reflects that FSH- and LH-secreting adenomas, while biochemically active, are frequently classified as clinically non-functioning (silent gonadotroph) tumors, as they do not typically produce a distinct hormonal syndrome. *14 reported having complications; however, the type of complications reported was 16, and the calculation of proportions was adjusted accordingly. Percent change in serum sodium was calculated by determining the change from the preoperative and postoperative values and dividing by the preoperative value and then multiplying by 100. Abbreviations: n=number of participants; sd=standard deviation; NA=missing data; DI=diabetes insipidus; SIADH=syndrome of inappropriate secretion of antidiuretic hormone; HTN=hypertension; ACTH=adrenocorticotropic hormone; FSH=follicle-stimulating hormone; LH=luteinizing hormone; CSF=cerebrospinal fluid; LHS=length of hospital stay.

Variable	n (%)
DI	7 (16.67)
SIADH	2 (4.76)
Both DI and SIADH	1 (2.38)
Normal	34 (80.95)
Female	24 (57.14)
Male	18 (42.86)
Non-smoker	39 (92.86)
Smoker	3 (7.14)
Diagnosis of HTN: No	33 (78.57)
Diagnosis of HTN: Yes	9 (21.43)
Visual symptoms: No	12 (33.33)
Visual symptoms: Yes	24 (66.67)
Headache: No	18 (51.43)
Headache: Yes	17 (48.57)
Pituitary apoplexy	1 (2.38)
Non-functioning	12 (28.57)
Functioning	18 (42.86)
Functionality: NA	12 (28.57)
ACTH-secreting	4 (22.22)
Prolactin-secreting	9 (50)
FSH-secreting	6 (33.33)
LH-secreting	3 (16.67)
Acromegaly	1 (5.50)
Cushing	5 (27.78)
Hypothyroidism	11 (61.11)
Panhypopituitarism	1 (5.50)
Macroadenoma	31 (73.81)
Microadenoma	10 (23.8)
Category of tumor size: NA	1 (2.38)
Initial surgery	35 (83.33)
Repeated surgery	7 (16.67)
Partial extent of resection	17 (40.48)
Total extent of resection	25 (59.52)
Endonasal surgery	16 (38.10)
Microscopic surgery	26 (61.90)
CSF leak	7 (43.75)
Panhypopituitarism	2 (12.50)
Infection	2 (12.50)
Pseudomeningocele	2 (12.50)
Hemorrhage	1 (6.25)
Other complications after surgery*	1 (6.25)
Invasions documented on imaging	22 (52.38)
Suprasellar extension	16 (72.73)
Cavernous sinus extension	6 (27.27)
Optic chiasma	1 (4.55)
Other brain structures	2 (9.10)
Percent change in serum sodium (mean±SD)	2.65±1.94
LHS in days (mean±SD)	9.15±8.90

No statistically significant differences were found in patients with DI compared with those who had SIADH or both DI and SIADH, and those without either complication (the normal group). These include differences in terms of the duration of length of hospital stay (LHS), preoperative serum sodium, preoperative prolactin (PRL), preoperative thyroid-stimulating hormone (TSH), and percent change in serum sodium. Mean LHS for the DI group was 8.00±5.48 days, which did not differ significantly from that of the SIADH group (8.00 days), but was significantly longer than that of the normal group (9.35±20.03 days) and much shorter than that of the group with both DI and SIADH (3.00 days; p=0.49). Similarly, there was no statistical significance in terms of preoperative serum sodium between the different groups, although the mean serum sodium values showed slight differences (mean: 137.33±1.63 mmol/L (DI), 139.00 mmol/L (SIADH), 138.71±3.59 mmol/L (normal), and 142.00 mmol/L (both DI and SIADH); p=0.55). However, similar findings were observed when comparing these variables across the different patient groups for preoperative PRL (mean: 201.03±126.53 mIU/L (DI), 175.00 mIU/L (SIADH), 1212.20±2582.68 mIU/L (normal), and 118.00 mIU/L (both DI and SIADH); p=0.74) and preoperative TSH levels in Table 2.

**Table 2:** Difference between patients' groups (DI, SIADH, both, and normal patients) according to LHS, preoperative sodium, PRL, and TSH levels, and percent change in serum sodium (N=43) Abbreviations: DI=diabetes insipidus; SIADH=syndrome of inappropriate secretion of antidiuretic hormone; LHS=length of hospital stay; PRL=prolactin; TSH=thyroid stimulating hormone. Continuous variables were compared using the Kruskal-Wallis test. The H statistic and corresponding p-values are reported. Continuous variables are presented as mean (standard deviation), and categorical variables as n (%). Dashes indicate values that could not be calculated due to insufficient data points for standard deviation: for the “Both DI and SIADH” group, this reflects the single-patient sample size (n=1); for the SIADH group, this reflects missing data for the specific variable in one of the two patients, leaving only one valid observation.

Variable	DI	SIADH	Normal	Both DI and SIADH	Kruskal-Wallis test	p
LHS	8 (5.48)	8.00	9.35 (20.03)	3.00	2.41	0.49
Preoperative sodium level	137.33 (1.63)	139.00	138.71 (3.59)	142.00	2.09	0.55
Preoperative level of serum PRL in mIU/L	201.03 (126.53)	175.00	1212.20(2582.68)	118.00	1.25	0.74
Pre-op level of TSH in uIU/L	1.11 (0.84)	1.49	1.52 (1.25)	0.68	0.82	0.85
Percent change in serum sodium	2.8 (2.85)	1.44	2.63 (1.84)	3.52	1.64	0.65

The baseline demographics, clinical features, and operative/postoperative findings for the presence of DI and SIADH are presented in Table 3. There were significant differences between the groups for patient age (n=7 versus all others, p=0.05); patients who developed DI were younger than non-DI patients (30.0±15.2 years versus 43.7±12.7 years). However, there were no significant differences in terms of gender distribution (female: 3/7 (42.9%) versus 21/35 (60.0%); p=0.68), BMI, smoking status (n=1 (14.3%) versus n=2 (5.7%); p=1.00), hypertension (n=0 (0.0%) versus n=9 (25.7%); p=0.31), visual symptoms (n=2 (28.6%) versus n=22 (62.9%); p=0.15), headache (n=2 (28.6%) versus n=15 (42.9%); p=0.71), pituitary apoplexy (n=0 (0.0%) versus n=1 (2.9%); p=0.75), tumor functionality (p=0.55), tumor size (p=0.87), surgery status (p=0.46), rate of resection (p=0.78), types of surgical techniques (p=0.89), and imaging evidence of invasion (p=0.89). No significant difference was found concerning the percentage change in serum Na^+^ levels or the LHS (p=0.42 and p=0.64, respectively) for both groups. However, different results were seen for postoperative complication rates. We detected that complications after surgery had happened more commonly after operations performed on patients with DI as compared to those who didn’t have this condition (n=6 out of seven patients with DI versus n=8 out of 35 patients without DI; p<0.01). Similarly, we noticed panhypopituitarism (2/7 vs. 0/35, p=0.02) and hypothyroidism (2/7 vs. 0/35, p=0.02) occurred significantly more frequently in patients with DI (Table 3).

**Table 3:** Relationship between DI/SIADH and patients' demographics, smoking status, comorbidities, associated symptoms, and length of hospital stay (LHS), preoperative, operative, and postoperative clinical data Note. The test used was the Chi-square test; * = independent sample t-test; ** = Mann–Whitney test. Abbreviations: n=number of participants; NA=missing data; DI=diabetes insipidus; SIADH=syndrome of inappropriate secretion of antidiuretic hormone; BMI=body mass index; HTN=hypertension; ACTH=adrenocorticotropic hormone; FSH=follicle-stimulating hormone; LH=luteinizing hormone; CSF=cerebrospinal fluid; LHS=length of hospital stay

Variable	No DI (n=35)	DI (n=7)	p	No SIADH (n=40)	SIADH (n=2)	p
Age at surgery*	43.70±12.70	30±15.20	0.05	41.60±13.50	38±29.70	0.89
Female	21 (60.00)	3 (42.86)	0.68	23(57.50)	1 (50.00)	1.00
Male	14 (40.00)	4 (57.14)	0.68	17(42.50)	1 (50.00)	1.00
BMI	29.98 (5.79)	25.65 (6.10)	0.29	29.48 (5.90)	23.60 (7.50)	0.18
Smoking	2 (5.71)	1 (14.29)	1.00	3 (7.5)	0 (0.00)	1.00
HTN	9 (25.71)	0 (0.0)	0.31	8 (20.00)	1 (50.00)	0.90
Visual symptoms	22 (62.86)	2 (28.57)	0.15	23 (57.50)	1 (50.00)	1.00
Headache	15 (42.86)	2 (28.57)	0.71	16 (40.00)	1 (50.00)	1.00
Pituitary apoplexy	1 (2.8)	0 (0.0)	0.75	1 (2.5)	0 (0.0)	1.00
Non-functioning	11 (31.43)	1 (14.29)	0.55	11(27.50)	1 (50.00)	0.62
Functioning	15 (42.86)	3 (42.86)	0.55	17 (42.50)	1 (50.00)	0.62
Functionality NA	9 (25.71)	3 (42.86)	0.55	12 (30.00)	0 (0.00)	0.62
ACTH-secreting	3 (8.57)	1 (14.29)	0.52	3 (7.50)	1 (50.00)	0.12
Prolactin-secreting	7 (20.00)	2 (28.57)	0.46	9 (22.50)	0 (0.00)	0.35
FSH-secreting	6 (17.14)	0 (0.00)	0.41	6 (15.00)	0 (0.00)	0.46
LH-secreting	3 (8.57)	0 (0.00)	0.53	3 (7.50)	0 (0.00)	0.56
Preoperative endocrinopathy	15 (42.86)	3 (42.86)	0.98	17 (42.50)	1 (50.00)	0.87
Macroadenoma	26 (74.29)	5 (71.43)	0.87	30 (75.00)	1 (50.00)	0.66
Microadenoma	8 (22.86)	2 (28.57)	0.87	9 (22.50)	1 (50.00)	0.66
Initial surgery	28 (80.00)	7 (100.00)	0.46	33 (82.50)	2 (100.00)	1.00
Repeated surgery	7 (20.00)	0 (0.00)	0.46	7 (17.50)	0 (0.00)	1.00
Partial resection	15 (42.86)	2 (28.57)	0.78	17 (42.50)	0 (0.00)	0.65
Total Resection	20 (57.14)	5 (71.43)	0.78	23 (57.50)	2 (100.00)	0.65
Endonasal surgery	14 (40.00)	2 (28.57)	0.89	15 (37.50)	1 (50.00)	1.00
Microscopic surgery	21 (60.00)	5 (71.43)	0.89	25 (62.50)	1 (50.00)	1.00
CSF leak	5 (14.29)	2 (28.57)	0.60	6 (32.50)	1 (50.00)	0.43
Panhypopituitarism	0(0.00)	2 (28.57)	0.02	2 (5.00)	0 (0.00)	1.00
Meningitis	0 (0.00)	2 (28.57)	0.02	2 (5.00)	0 (0.00)	1.00
Pseudo-meningocele	2 (5.71)	0 (0.00)	0.72	2 (5.00)	0 (0.00)	0.92
Hemorrhage	1 (2.86)	0 (0.00)	0.81	1 (2.50)	0 (0.00)	0.95
Other complications after surgery	4 (11.43)	1 (14.28)	0.89	4 (10.00)	1 (50.00)	0.23
Invasions documented on imaging	19 (54.29)	3 (42.86)	0.89	21 (52.50)	1 (50.00)	1.00
Percent change in serum sodium**	2.59 (1.83)	2.90 (2.61)	0.42	2.65 (1.98)	2.48 (1.47)	0.11
LHS**	9.29 (9.81)	7 (5.24)	0.64	9.15 (9.42)	5.50 (3.54)	0.57

We conducted multivariable logistic regression analyses for the identification of independent predictive factors for post-operative DI. Age at surgery (OR=0.92, p=0.153), the absence of postoperative complications (OR=0.11, p=0.119), and absence of panhypopituitarism (p=0.996) were not independently associated with developing DI. Our regression model consisted of data from 42 patients with a Tjur’s R² value of 0.625, suggesting a moderate discriminative ability of our model (Table 4).

**Table 4:** Multiple logistic regression results Abbreviations: sd=standard deviation; CI=confidence interval

	Diabetes Insipidus (DI)				
Predictors	Odds ratios	Standard error	CI	Z-Statistic	P
(Intercept)	441949654580134592.00	2713306586237149118464.00	0.00 - Inf	0.01	0.995
Age when surgery was done	0.92	0.05	0.00 - Inf	-1.43	0.153
Complications: No	0.11	0.16	0.00 - Inf	-1.56	0.119
Panhypopituitarism: No	0.00	0.00	0.00 - Inf	-0.00	0.996
Observations	42				
R^2^ Tjur	0.625				

## Discussion

Symptomatic hyponatremia and DI have proven to be significant contributors to readmission, medical expenses, and morbidity. Hence, diagnosing and treating these disorders before discharge is preferred. A strategy to identify patients at risk of postoperative electrolyte abnormalities would aid in planning and focused surveillance of high-risk patients in the ICU. Hence, in this study, we investigated the viability of predictors for the development of postoperative DI and SIADH in a retrospective registry of all patients who underwent TSS for pituitary adenoma at our institution between 2010 and 2022.

In total, 16.68% of patients were diagnosed with DI during their hospital stay and follow-up period, which is comparable to the results reported by Nemergut et al. [19]. Moreover, this number is notably lower than the incidences of 38.5% and 27.8% reported by Kristof et al. [20]. and Faltado et al. [13], respectively. Unlike our study, these studies included more invasive surgical approaches, such as craniotomy, and different pathologies of pituitary diseases, including Rathké's cleft cyst and craniopharyngiomas, which typically damage the neurohypophysis, requiring more extensive surgical intervention that could have resulted in the higher incidence of DI [15]. Furthermore, the diagnostic criteria used in these studies were notably less restrictive than ours.

Our study revealed a borderline association between age and the subsequent development of DI. Ajlan et al. reported a stronger association [21]. In contrast, Kristof et al. attributed the increased occurrence of DI in younger patients to the more aggressive surgical procedures commonly performed in this population, involving extensive manipulation or removal of the pituitary gland [20]. Younger patients have a longer life expectancy [22]. However, the mean age of our participants was 44 years, and only three patients were aged <18 years, highlighting the need for further research, specifically targeting younger age groups.

Our study found no correlation between the risk of DI in non-secretory and secretory adenomas, nor among the underlying functional subtypes. These findings are consistent with the results of previous studies, which also concluded the same finding [14,17]. Moreover, previous studies have reported inconsistent findings regarding the correlation between tumor size and DI. Some studies have indicated that patients with macroadenomas are more likely to develop postoperative DI [14,21]. Similar to a few other studies, we observed no discernible pattern between the development of DI and tumor size. Although larger tumors can compress and disrupt the normal behavior of the pituitary gland, they are easier to remove than smaller adenomas, which require extensive exploration. In this study, most procedures (72.8%) were performed for macroadenomas, which limited the generalizability of the findings. However, these results provide an important understanding of the outcomes in this specific subset of patients [11,13].

Regarding postoperative complications, studies have reported a decreased incidence of CSF leaks in recent years. This may be attributed to increased awareness and continuous advancements in skull base reconstruction techniques [23,24]. However, CSF leaks were the most prevalent surgical complication among our participants, and in contrast to previous studies, no relationship between DI and CSF leaks was found in this study [14,21]. Interestingly, unlike previous research, almost half of the DI group in our study had hypopituitarism, including central hypothyroidism after surgery, showing a statistically significant relationship with DI. Hence, aiming for optimal pituitary and thyroid hormone levels postoperatively may improve patient outcomes. Nayak et al. suggested a high prevalence of undertreatment in patients with central hypothyroidism [14]. Comprehensive care provided by neurosurgeons and endocrinologists is necessary to improve surgical outcomes and to preserve and restore pituitary function.

The incidence of SIADH in our sample was 4.8%, markedly lower than the 14% [11] and 6.3% [12] reported in the previous studies. Saldarriaga et al. [11] included pediatric patients, which may explain the discrepancy in our results. Although previous studies have extensively discussed the risk factors for hyponatremia after surgery, research specifically focusing on the preoperative risk factors for SIADH is limited. Preoperative hyponatremia has been found to play a major role in predicting SIADH [11,12]. In our study, two patients were found to have hyponatremia; both patients’ sodium levels were corrected by the second postoperative day, and neither developed SIADH during the postoperative period. Therefore, no correlation was found between preoperative sodium levels and SIADH. Among the four patients diagnosed with ACTH-secreting adenomas, one eventually developed SIADH. However, the relationship was not statistically significant. This aligns with the findings of a previous study. In contrast, ACTH deficiency before surgery showed a significant association with hyponatremia, possibly because ADH is stimulated by ACTH deficiency, and hyponatremia can be an initial sign of cortisol deficiency [25].

One of the earliest documented and most well-understood effects of nicotine is its ability to directly stimulate the release of ADH, leading to elevated urinary osmolality and reduced free water clearance [26]. Therefore, we investigated the effect of smoking on the incidence of electrolyte abnormalities after TSS; however, neither DI nor SIADH was associated with smoking. We also examined the influence of chronic hypertension on the occurrence of DI and SIADH; however, no such correlation was observed in this study. Notably, no prior study has investigated the relationship between smoking or chronic hypertension and SIADH or DI after PS.

Electrolyte imbalance is a serious concern because it requires continuous monitoring and prolonged hospital and ICU stays. However, in our study, patients with DI or SIADH showed no significant variation in LHS compared with normal patients [27]. Our patients were monitored closely as inpatients for a mean of 8.83 (8.99) days, compared with four or six days in the previous study [21]. This difference may be attributed to protocols adopted by different institutions for electrolyte disturbance management.

Our study had some limitations. First, our study had a small sample size due to the low number of pituitary cases in the region despite being performed at a referral hospital for all pituitary cases in the western region of Saudi Arabia. However, a previous study reported findings based on a small number of patients, supporting the significance of our study. Second, two patients were diagnosed with SIADH (4.8%) [28]. Due to this low rate of events, the multivariable logistic regression model for this state cannot be developed effectively, and any results observed has to be treated with extreme caution. This study may have inadequate statistical power to observe clinically significant predictors for states of DI and SIADH. Thirdly, our study was conducted in a single center; thus, an "institutional or center bias" could be a potential source of variability as far as surgical techniques are concerned. Lastly, this study did not make a distinction between transient and permanent forms of DI; in addition, volumetric analysis of tumor dimensions was not performed. Considering these factors, our results may be perceived as an initial contribution to the regional literature, and further prospective studies with larger study populations, including multiple centers, would be necessary to sufficiently validate these results statistically.

## Conclusions

Postoperative DI was observed more often than SIADH in patients with pituitary adenomas who underwent transsphenoidal surgery in this group. While young age and postoperative endocrine dysfunction, namely panhypopituitarism, including central hypothyroidism, were predictors of DI according to the univariate analysis, there were no independent preoperative or perioperative predictors of either DI or SIADH in the multivariable model. Such results underline the complex nature of postoperative disorders of water-electrolyte metabolism and stress the current inability to use existing clinical data to perform reliable risk stratification. Considering the potential complications related to the postoperative disturbances in sodium level, the importance of close monitoring of patients' fluid balance, urine output, and sodium level should be stressed in all patients who undergo transsphenoidal surgery. Cooperation of neurosurgeons and endocrinologists might be useful for the timely identification and treatment of such postoperative complications, thus improving patients' outcomes. Future large prospective multicenter studies will be helpful in validating these results and constructing predictive models.
